# Color constancy for daylight illumination changes in anomalous trichromats and dichromats

**DOI:** 10.1364/JOSAA.479961

**Published:** 2023-03-01

**Authors:** Stacey Aston, Gabriele Jordan, Anya Hurlbert

**Affiliations:** 1 Department of Psychology, Durham University, Durham DH1 3LE, UK; 2 Centre for Transformative Neuroscience and Institute of Biosciences, Newcastle University, Newcastle upon Tyne NE2 4HH, UK; 3 School of Psychology, Newcastle University, Newcastle upon Tyne NE2 4DR, UK

## Abstract

Color constancy is the perceptual stability of surface colors under temporal changes in the illumination spectrum. The illumination discrimination task (IDT) reveals worse discrimination for “bluer” illumination changes in normal-trichromatic observers (changes towards cooler color temperatures on the daylight chromaticity locus), indicating greater stability of scene colors or better color constancy, compared with illumination changes in other chromatic directions. Here, we compare the performance of individuals with X-linked color-vision deficiencies (CVDs) to normal trichromats on the IDT performed in an immersive setting with a real scene illuminated by spectrally tunable LED lamps. We determine discrimination thresholds for illumination changes relative to a reference illumination (D65) in four chromatic directions, roughly parallel and orthogonal to the daylight locus. We find, using both a standard CIELUV metric and a cone-contrast metric tailored to distinct CVD types, that discrimination thresholds for daylight changes do not differ between normal trichromats and CVD types, including dichromats and anomalous trichromats, but thresholds for atypical illuminations do differ. This result extends a previous report of illumination discrimination ability in dichromats for simulated daylight changes in images. In addition, using the cone-contrast metric to compare thresholds for bluer and yellower daylight changes with those for unnatural redder and greener changes, we suggest that reduced sensitivity to daylight changes is weakly preserved in X-linked CVDs.

## 1. INTRODUCTION

In broad terms, color constancy describes the ability of human observers to assess object color as stable across changes in the incident illumination (for reviews, see [1–5]). It is an important feature of human color cognition that supports the use of color for object recognition and for object description in communication.

What color constancy means in terms of the individual’s perceptual experience, though, depends not only on the individual, but also on how it is measured. It is well known that when asked to match exactly the color appearance of a fixed surface under changing illumination, individuals may indicate that the surface has changed in hue, saturation, or lightness, yet nonetheless report that the surface has not materially changed [6,7]. The extent to which such a dissociation occurs depends on the framing of the task as well as the experimental conditions, such as exposure duration and the particular surface reflectances and illumination spectra tested [8,9]. The illumination discrimination task (IDT) [10,11] provides a measure of color constancy at the appearance level only, by determining the magnitude of illumination change, which causes a just-noticeable change in the scene appearance. The participant is not required to distinguish between different types of change, only to detect any change in the scene. In this sense, the illumination discrimination threshold provides a lower bound for color constancy; below this threshold, by definition, any effect of the illumination change on surface color appearance will be undetectable.

Here we explore the influence of differences in the individual’s sensory apparatus—specifically their complement of retinal cone types—on color constancy at the appearance level. Previous measurements of illumination discrimination thresholds for global illumination changes show an asymmetric daylight bias in individuals with normal-trichromatic color vision (hereafter referred to as normal trichromats). Thresholds are consistently higher for global illumination changes towards “bluer” daylights than towards unnatural “redder” or “greener” illuminations, but not always higher for changes towards “yellower” daylights [10–13], all changes defined relative to neutral illuminations. Higher discrimination thresholds, or reduced sensitivity, to “bluer” daylight changes in illumination for normal trichromats imply that the human visual system is more tolerant of these types of illumination changes. It is hypothesized that this bias comes about from an internalization (either innate or learned) of the statistics of daylight illuminations that optimizes color constancy mechanisms at the appearance level for the natural environment [10,11]. While daylights more commonly fall on the “yellower” end of the Planckian locus, they tend to be more saturated on the “bluer” end [14,15], suggesting that the human visual system better tolerates, or is less sensitive to, transitions between naturally occurring illuminations.

While the majority of studies have investigated color constancy in normal trichromats, only a handful of studies have explored color constancy in individuals with color-vision deficiencies (CVDs) [16–20]. Studying color constancy in CVDs is important for understanding how an individual’s perceptual experiences and cognitive interactions with the world may be influenced by differences at the lowest level of sensory processing.

In normal trichromats, the retina houses three types of cone cells that may be differentiated by their peak spectral sensitivities: the long- (L), middle- (M), and short- (S) wavelength sensitive cones. However, approximately 8% of the male European Caucasian population [21] possess one of several distinct types of X-linked CVD, having either dichromacy, in which either the M or L cone type is lacking (deuteranopia or protanopia, respectively), or anomalous trichromacy, in which either the M or L cone spectral sensitivity is spectrally shifted (deuteranomaly or protanomaly, respectively). Both forms of anomalous trichromacy result in the peaks of the two middle-to long-wavelength cone sensitivities being closer together than normal, causing reduced discrimination along the cardinal L–M axis. Dichromacy causes a collapse in discrimination along this axis. Yet in both normal and anomalous color vision, distinct varieties of L and M cone class photopigments, including L/M hybrids, may occur. These varieties differ in amino acid sequences encoded at key sites, which in turn confer differences in the cones’ wavelengths of peak spectral sensitivity. Even in normal trichromacy, the separation between the L and M cones’ peak sensitivities may vary, between 13 and 29 nm by estimates based on genetic sequencing [22,23].

Previously, Alvaro *et al*. [16] measured illumination discrimination thresholds in deuteranopes and protanopes by simulating “bluer” and “yellower” daylight changes on hyperspectral images of natural scenes. By comparing CVD thresholds to those of normal trichromats, they concluded that illumination discrimination ability, and hence appearance level color constancy, was preserved along the daylight axis for dichromats. We show that this result extends to illumination discrimination thresholds measured for real scenes and real illumination changes in both dichromats and anomalous trichromats. We go further to measure thresholds for discriminating real illumination changes in atypical “redder” and “greener” directions, and use a cone-contrast metric tailored to different cone complements to compare performance across chromatic directions within different CVD types.

## 2. METHODS

The methods used in this experiment were the same as those described in a previous paper [10] with only minor changes to the procedure but no changes to the equipment.

### A. Methods Overview

**Fig. 1.**
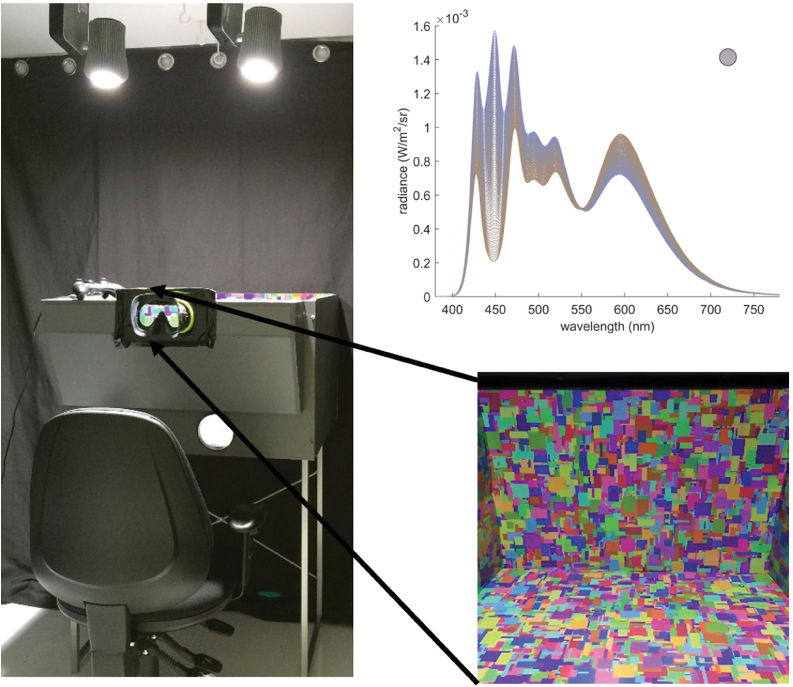
Experimental setup. Participants positioned their head against the goggles, restricting their view to inside the stimulus box, shown in inset. The box was illuminated by two spectrally tunable LED luminaires, which also provided the general room illumination. Upper right, sample illumination spectra varying in the “bluer” and “yellower” directions away from the neutral (D65) reference illumination.

Twenty-two normal trichromats and 22 CVD participants completed an IDT. In the task, as previously reported [10], participants were seated in front of a Mondrian-papered box (the scene) (Fig. 1). The box was lit from above by two 10-channel spectrally tunable LED lamps. The participants, who were also immersed in the illumination, viewed the box through a porthole. On each trial, the scene was first lit by a reference illumination of neutral chromaticity and then two comparisons, with each illumination separated by a short period of darkness. One of the comparison illuminations (the target) was identical to the reference; the other comparison (the test) varied from the reference along one of four axes, a “bluer”/“yellower” axis resembling natural illumination changes and a “redder”/“greener” axis for atypical illumination changes. The participant was instructed to indicate, using a button press, the comparison that most closely matched the reference illumination. The “bluer” and “yellower” directions of illumination change were parameterized to fall on the Planckian locus, closely following the daylight chromaticity locus, while the “redder” and “greener” illuminations fell along an orthogonal locus. For each direction of illumination change, there were 50 alternative test illuminations, spaced approximately one $\Delta E_{u^{*}v^{*}}$ apart such that one nominal step along each axis corresponded roughly to one perceptual step in CIELUV for normal trichromats. Interleaved staircases determined the test illumination to be shown on each trial to establish independent illumination discrimination thresholds for each of the four directions of illumination change. Further details are provided below.

### B. Participants

We recruited 22 CVD observers to take part in the experiment: three deuteranopes (all male, 19–22 years); five protanopes (all male, 19–29 years); 11 deuteranomalous individuals (one female, 19–24 years); and three protanomalous individuals (one female, 20–26 years). Each participant’s type of CVD was characterized using Ishihara plates [38 plate edition, Kanehara Shuppan Co., Ltd. (now known as Kanehara Trading Inc.), Tokyo, Japan], the Farnsworth-Munsell 100-Hue test (Munsell Color, X-Rite, Inc.), and an Oculus anomaloscope (OCULUS HMC-Anomaloskop, OCULUS, Germany). We also recruited 22 normal-trichromat participants (15 female, 18–31 years) to serve as a comparison group. Ishihara plates and the Farnsworth–Munsell 100-hue test confirmed that each participant had normal-trichromatic color vision. All participants had normal or corrected-to-normal visual acuity and received cash compensation for their time.

Ethical approval for the study was received from the Newcastle University Ethics Board. Written consent was received from all participants prior to participation in the study.

### C. Scene

The scene consisted of a box (dimensions: H, 45 cm; W, 77.5 cm; D, 64.5 cm) interiorly lined with a matte printed Mondrian (Fig. 1), and illuminated from above through its open top. Participants viewed the scene through head-stabilizing goggles mounted on a porthole designed to restrict their view to the interior back, sides, and bottom of the box, ensuring that the Mondrian paper covered their full visual field. The viewing distance from the participant’s eyes to the back of the box was 81 cm. Both the scene and the participant were immersed in the illumination (the only source of light in the room).

The Mondrian paper was designed and printed specifically for the experiment. It consisted of 10s of 1000s of small rectangular colored patches, varying in height and width from 2 to 42 mm (0.14° to 2.97° of visual angle), randomly placed and overlapping to ensure complete coverage of the paper. The average chromaticity of the 24 unique surface reflectances used to generate the patches was approximately CIE (x,y)=(0.33,0.33) under a hypothetical equal energy light source. The space-averaged surface reflectance of the Mondrian paper was approximately equal at all wavelengths. Surface reflectance functions for each patch and further details of the Mondrian generation and measurements are provided in [10].

### D. Illuminations

The illuminations were produced using two 10-channel (nine unique) spectrally tunable LED lamps (HI-LED Prototype I luminaires; produced by the Catalonia Institute for Energy Research, Barcelona, Spain, as prototypes for the EU FP7-funded HI-LED project). Spectra were obtained by optimizing the weights on the individual LED channels to produce the smoothest spectrum possible with the desired lux and chromaticity, using custom spectral fitting procedures implemented in MATLAB [11,24]. The spectral power distributions of the resulting spectra were subsequently measured, and these measurements were used in all further calculations.

The chromaticity coordinates of the reference illumination (D65) were (x,y)=(0.31,0.33) in CIE 1931 space. The test illuminations were generated such that they varied systematically away from the chromaticity of the reference illumination in one of four distinct chromatic directions. We label these, for simplicity and not to imply specific perceptual phenomena, “bluer,” “yellower,” “redder,” and “greener” (Fig. 1). (We hereafter drop the quotation marks on the labels.)

Bluer and yellower test illuminations were parameterized to fall along the CIE daylight locus (close to the Planckian locus, defined in the CIE xy chromaticity plane as $y=2.87x-3x^{2}-0.275$ [25]), approximating natural illumination changes. Redder and greener test illuminations were parameterized to follow the constant correlated color temperature (CCT) line through the reference illumination chromaticity, creating atypical illumination changes.

The chromaticities of the 50 illuminations in each test set were spaced approximately one $\Delta E_{u^{*}v^{*}}$ apart, calculated with the neutral reference illumination as the fixed white point. Their illuminance levels were held constant such that under each illumination the luminance of a white polymer calibration tile placed flush against the back wall of the stimulus box, orthogonal to the viewing direction, was $Y=50\;cd/m^{2}$ . It is important to note here that, following previous studies [10–13], we chose CIELUV, a normally perceptually uniform space, to characterize the distances between the alternative illumination spectra used as stimuli, yet having the full spectral power distributions of each illumination allows us to calculate distances and discrimination thresholds in any other desired color space.

The LED lamps were spectrally calibrated by measuring the outputs of the individual LED channels at maximum power, using a CS2000 Konica Minolta spectroradiometer (Konica Minolta, Nieuwegein, Netherlands) to take radiance measurements from a polymer white reflectance tile in fixed position under sole illumination from each channel. The full procedure is described in [10].

### E. Procedure

**Fig. 2.**
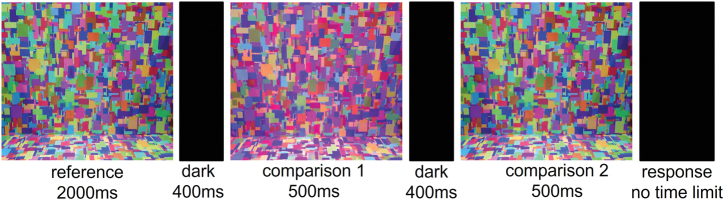
Timeline for each trial in the IDT. Colors shown in this figure are illustrative and approximate only the actual experimental stimuli.

At the start of the experiment, participants read the standardized instructions and were permitted to ask questions. All participants then received the same verbal instructions: “On each trial, you will see the reference illumination followed by two comparison illuminations. You will use the gaming pad to indicate which of the two comparison illuminations most closely matched the reference.” Participants were then dark adapted for two min before starting the task. On each trial, the reference illumination was visible for 2000 ms. The illumination was switched off for a dark interval of 400 ms. The two comparison illuminations were then displayed for 500 ms each, separated by a dark interval of 400 ms (Fig. 2). The interval in which the comparison illumination equaled the reference illumination was randomized across trials.

Thresholds for each illumination-change direction were found using a one-up, three-down, transformed, and weighted staircase procedure (step sizes of one, two, or three depending on the preceding run of trials [26]). Staircases started at a random nominal step between 10 and 20, 20 and 30, 30 and 40, or 40 and 50 steps away from the reference and terminated after six reversals. The starting step interval was determined separately for each direction of change based on a set of trials at the start of the task, as follows. A participant was first presented with five trials in which the test illumination was 20 nominal steps from the reference. If participants responded correctly to at least four of these trials, staircases started at random points in the interval 10 to 20 nominal steps away. Otherwise, they were presented with five trials where the test was 30 nominal steps away, and so on. No participant failed to get at least four trials correct by the limit of the five tests being 40 steps away.

Three interleaved staircases were completed for each direction of illumination change. Staircases for the four illumination-change directions were also interleaved, for 12 interleaved staircases in total. Participants were told that they could take as long as they needed to respond and that they could take a break at any time during the experiment (by remembering their response, but not entering it until they were ready to continue). Participants were required to take a mandatory break after every 100 trials. This break could last for as long as the participant desired. Participants did not leave the dark experimental room during breaks.

### F. Luminance Calculations

The illuminations were designed such that their illuminance levels were held stable across the experiment for normal trichromat observers, by keeping constant the luminance of a fixed calibration tile under that sole illumination. Thus, chromatic differences only would drive discrimination. This design does not guarantee that luminance would be constant for the different types of CVD observers, or indeed for each individual normal trichromat observer, given the known variations in L and M cone sensitivities [22,23]. To demonstrate that any residual luminance changes between stimuli would not contribute to performance for any nonstandard observer, we calculated the maximum change in luminance observable by distinct color-vision types, creating tailored luminance functions covering the range of L and M peak wavelength separations and their combinations for normal and anomalous trichromacy and dichromacy [22,23,27]. The resulting maximum luminance changes are shown in Table 1, Appendix A.

For all CVD types and chromatic directions, the maximum change in luminance over the entire set of 50 stimuli is very small, with the largest difference between successive stimuli equivalent to a change of $0.12\;cd/m^{2}$ in the luminance of the fixed calibration tile, or a Weber fraction of 0.0024. This difference would occur for protanopic individuals with peak wavelengths of [530, 530, 420] for their nominal L, M and S cone types. Importantly, there is no difference between CVD types in the variation of maximum illuminance differences across chromatic directions [ F(12,20)=0.319 ; p=0.977 ]. Any effect of illuminance differences on discrimination performance between chromatic directions would therefore be the same across all CVD types.

### G. Data Analysis

As noted above, thresholds for illumination discrimination ability along each direction of change may be calculated in any metric, given the physical specification of the illumination spectra. In previous studies using the same experimental paradigm [10–13,16], thresholds have been computed in CIELUV [28], a space historically designated for lighting and approximately perceptually uniform for normal trichromats. Alvaro *et al*. [16] also calculated thresholds as differences in CCTs along the CIE daylight chromaticity locus (in ${MK}^{-1}$ ), and found for both metrics that patterns with respect to chromatic directions were similar and that discrimination thresholds were comparable for both dichromats and normal trichromats.

To compare performance between participant groups, it is important to use the same metric to define differences between stimuli, and in order to compare results here with previous results, we therefore use CIELUV as the first metric for threshold calculations. CIELUV will not, in general, be perceptually uniform for other color-vision types. To compare thresholds between chromatic directions within participants, therefore, we also use a second metric, the contrast signal recorded by the cones. Because the L and M cone spectral sensitivities will vary between and possibly within types of trichromacy and dichromacy, so too will the magnitude of the cone-contrast signal recorded for any particular change in illumination spectrum. Cone-contrast units, though, will be equivalent across cone types, by definition.

For both methods, we map each nominal value (1–50 for the 50 test illuminations along each direction of change) to the difference between the corresponding test and reference measured illumination spectra, computed in the appropriate metric.

For the CIELUV metric [28], we calculate each difference by computing XYZ tristimulus values for each of the measured illumination spectral power distributions using the CIE 2006 color-matching functions [29]. The XYZ values are transformed to CIELUV using the XYZ of the reference illumination as the white point while holding luminance fixed at $50\;cd/m^{2}$ for all illuminations. The difference is then defined as

$$\Delta E_{u^{*}v^{*}}=\sqrt{{\left(L_{t}^{*}-L_{r}^{*}\right)}^{2}+{\left(u_{t}^{*}-u_{r}^{*}\right)}^{2}+{\left(v_{t}^{*}-v_{r}^{*}\right)}^{2}},$$

where $L_{r}^{*}u_{r}^{*}v_{r}^{*}$ and $L_{t}^{*}u_{t}^{*}v_{t}^{*}$ are the reference and test illuminations expressed in CIELUV, respectively. Note that by holding luminance constant in these calculations, the term ${\left(L_{t}^{*}-L_{r}^{*}\right)}^{2}=0$ , and therefore $\Delta E_{u^{*}v^{*}}$ captures only chromatic differences in the illuminations. Finally, we take the threshold as the mean over the last two reversals from each of the three interleaved staircases.

For the cone-contrast metric, for each nominal step in each direction of change, we calculate the total cone contrast ( ΔCC ) between the LMS tristimulus values for the measured test and reference illuminations as

$$\Delta CC=\sqrt{{\left(\frac{L_{t}-L_{r}}{L_{r}}\right)}^{2}+{\left(\frac{M_{t}-M_{r}}{M_{r}}\right)}^{2}+{\left(\frac{S_{t}-S_{r}}{S_{r}}\right)}^{2},}$$

where $L_{r}M_{r}S_{r}$ and $L_{t}M_{t}S_{t}$ are the cone responses to the reference and test illuminations, respectively, computed from the product of the cone spectral sensitivity curves and the illumination spectral power distributions.

Ideally, ΔCC would be calculated separately for each observer type depending on their respective cone complements. Because we do not know the varieties of L and/or M cone classes for each individual, we instead calculate two sets of ΔCC values for each color-vision type, covering the range of L and M peak wavelength separations and their possible combinations [22,23]. Cone fundamentals are calculated for each peak sensitivity using the Stockman–Sharpe template [30]. For protanopes, who lack the L cone, and express a single functional opsin of the M class (so that L=M in the above formula) the latter is assigned a peak sensitivity of either 530 nm or 536 nm, i.e., [ $L_{peak},\;M_{peak}$ ] of [530, 530] or [536, 536]. For deuteranopes, lacking the M cone, the L cone peak is either 549 or 559. Deuteranomalous types are assigned L and M peak combinations of either [559, 549] or [559, 556.5], and protanomalous types either [536, 530] or [536, 533]. For normal trichromats, the combinations are [559, 530] or [549, 536]. In all cases the S cone peak sensitivity is assigned 420 nm.

We calculated an additional set of ΔCC values using the DeMarco cone fundamentals [27] that include spectral sensitivity curves for anomalous L and M cones denoted as $L^{\prime }$ and $M^{\prime }$ . The peaks of the five DeMarco *et al*. (1992) sensitivity functions are 440 nm, 543 nm, 566 nm, 558 nm, and 553 nm for the $S,M,L,M^{\prime }$ , and $L^{\prime }$ cone types, respectively. For the deuteranopic participants we use only the L and S responses; for protanopic participants, only M and S ; for deuteranomalous or protanomalous participants, $L,\;M^{\prime }$ , and S or $L^{\prime },\;M$ , and S , respectively.

Once all nominal values have been mapped to a ΔCC , thresholds are then (as before) computed as the mean over the last two reversals from each of the three interleaved staircases.

In the text and figures presented in the results and discussion, all data are presented as means over participants and associated standard errors. Error bars are always one standard error. For ANOVA analyses, if the assumption of sphericity was violated an appropriate correction was performed depending on the value of ε (Greenhouse–Geisser if ε≤0.75 ; Huynd–Feldt if ε>0.75 ). Where pairwise comparisons and simple main effects are reported, p values have been corrected for multiple comparisons by applying a suitable Bonferroni correction. For example, where simple main effects analyses are used to follow up the finding of a significant interaction in a two-way ANOVA with factors A and B, the p values of the one-way ANOVAs for factor A are multiplied by the number of levels for factor B and vice versa. Similarly, when a one-way ANOVA whose factor has m levels is followed up with pairwise comparisons, the p values resulting from these comparisons are multiplied by m(m−1)/2 , the number of possible pairwise comparisons.

## 3. RESULTS AND DISCUSSION

### A. CIELUV Thresholds Analysis

**Fig. 3.**
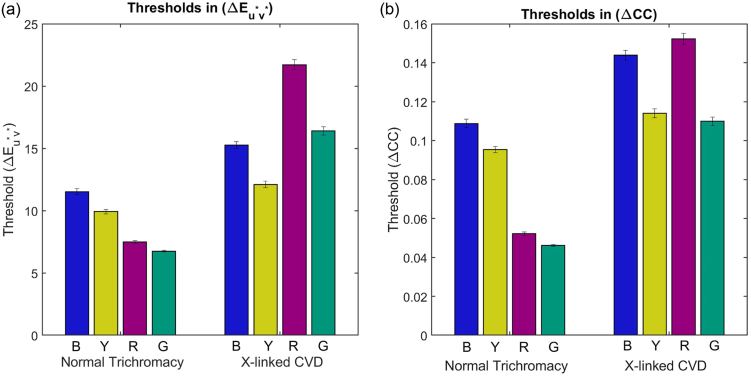
(a) Illumination discrimination thresholds across the four directions of illumination change (B, blue; Y, yellow; R, red; G, green) split by color-vision type (normal trichromat versus X-linked CVD) expressed in CIELUV ( $\Delta E_{u^{*}v^{*}}$ ). (b) Same data as in (a) but now with thresholds expressed in terms of total cone contrast ( ΔCC ). Error bars are ±1SEM .

To quantify differences in illumination discrimination thresholds between normal trichromats and X-linked CVD participants, we first performed a one-way repeated measures ANOVA on the thresholds expressed in CIELUV (where one threshold unit roughly represents one $\Delta E_{u^{*}v^{*}}$ with a between groups factor of color-vision type (two levels: normal trichromat versus X-linked CVD) and a within groups factor of direction of illumination change (four levels: bluer, yellower, redder, greener). We find a significant interaction effect of color-vision type and direction of illumination change on thresholds [ F(3,126)=18.43 , p<.001 ; Fig. 3(a)].

Simple main effects of color-vision type reveal that the interaction effect is driven by significantly larger thresholds for the CVD group in the redder [ t(42)=−7.33 , p<.001 ] and greener [ t(42)=−5.85 , p<.001 ] directions of illumination change, but no difference across groups for the bluer [ t(42)=−2.28 , p=.113 ] and yellower [ t(42)=−1.47 , p=.599 ] directions. Subsequently, the simple main effect of the direction of illumination change is significant for both groups [normal trichromats: F(3,63)=13.84 , p<.001 ; CVDs: F(3,63)=12.10 , p<.001 ], but the pattern of thresholds differs. In the normal-trichromat group, thresholds are significantly higher in the bluer and yellower directions of change than in the redder and greener directions ( p<.05 in all cases). In the CVD group, thresholds are significantly lower in the bluer and yellower direction than in the redder direction and significantly lower in the yellower than in the greener direction ( p<.003 in all cases).

### B. Interim Discussion: CVD Versus Normal Trichromacy

Alvaro *et al*. [16] reported no difference in illumination discrimination thresholds, expressed as both CIELUV and CCT differences, for simulated daylight changes between normal trichromats and dichromats, in scenes rendered on a monitor. Here, we show that this result extends to a situation in which participants are viewing a real scene under real illuminations; to a larger group of CVD observers that includes anomalous trichromats as well as dichromats, and in comparison with a larger set of illuminations, including nondaylight illumination changes. In sum, these results show that discrimination of illuminations, and, hence, the appearance level color constancy, for daylight changes is preserved in X-linked CVD. This is likely a contributing factor to anomalous L and M cone opsins surviving natural selection; color constancy at the appearance level is preserved for the types of natural illumination changes under which humans have evolved.

Significantly higher thresholds in normal trichromats for bluer and yellower compared to redder and greener illumination changes replicate several previous reports of a daylight bias in illumination discrimination ability, though we note that the bias here is symmetric on the daylight axis, with thresholds for both bluer and yellower directions of change significantly larger than redder and greener directions rather than only an elevation of blue thresholds, as has been reported previously [10–13]. However, while there is no difference in thresholds for bluer and yellower changes between normal trichromats and CVDs, thresholds for redder and greener directions are higher in CVD observers than in normal trichromats. If illumination discrimination thresholds provide a measure of the lower bound for appearance level color constancy, these results imply that CVD observers have superior constancy to normal trichromats for atypical illumination changes, when evaluated by the same objective metric (CIELUV). For CVD observers, scene appearance will remain more constant under atypical illumination changes than it will for normal trichromats.

It does not follow, though, that individual CVD observers will have superior constancy for atypical versus typical illumination changes, i.e., that they will possess the opposite of a daylight bias. Although for CVD observers yellower thresholds are significantly lower than redder and greener thresholds, and bluer thresholds significantly lower than redder thresholds, this has been demonstrated only for the CIELUV metric, which does not guarantee that unit differences will be perceptually equal across color space for individuals whose cone complements differ from standard trichromacy. Significant differences in CIELUV might not be significantly perceptually different for individual CVD observers.

Therefore, we turn to the lowest level at which a change in response to spectrally varying stimuli would be registered at the cones. To compare thresholds between chromatic directions within participants, we use the total cone-contrast metric, or ΔCC , by calculating the contrast signal between the reference and test illuminations as recorded by the cones.

### C. Cone-Contrast Threshold Analysis

As described above, we calculated three sets of cone-contrast measures for each color-vision type: normal-trichromatic (NT), protanopic (PD), deuteranopic (DD), protanomalous, (PA), and deuteranomalous (DA). Each set assumes a different complement of L and M cone varieties, with different combinations of peak sensitivity wavelengths. The significant findings are essentially the same for all combinations of these sets. We report the details for one combination below, in which the [ $L_{peak},\;M_{peak}$ ] the wavelengths are NT: [530, 559]; PD: [530, 530]; DD: [549, 549]; PA: [530, 536]; and DA: [549, 559], and give details of two additional combinations in Tables 2 and 3, Appendix A.

Repeating the analysis described above after swapping out the dependent variable for the ΔCC threshold measure, instead of CIELUV, we again find a significant interaction effect of color-vision type and direction of illumination change on thresholds [ F(3,126)=11.703 , p<.001 ; Fig. 3(b)]. Simple main effects of color-vision type show that the interaction effect is driven by the same differences as before: significantly larger thresholds for the CVD group in the redder [ t(42)=−7.29 , p<.001 ] and greener [ t(42)=−5.82 , p<.001 ] directions but not bluer [ t(42)=−2.2 , p=.134 ] and yellower [ t(42)=−1.42 , p=.657 ].

The simple main effect of the direction of illumination change remains significant for both groups [normal trichromats: F(3,63)=31.9 , p<.001 ; CVDs: F(3,63)=5.7 , p=.011 ]. In the normal-trichromat group, thresholds are still significantly higher in the bluer and yellower directions of change than in the redder and greener directions of change ( p<.001 in all cases). However, in the CVD group, thresholds are significantly higher in the redder direction than the greener direction ( p=.048 ) and tend to be higher in the bluer direction than the greener direction ( p=.07 ).

In summary, when we quantify thresholds in terms of the total cone contrast between the reference and test illuminations that would be elicited from the particular cone complements of observers with different color-vision types, all of the results from analyzing thresholds in CIELUV hold, except there is no longer the opposite of a daylight bias in CVDs. Instead, there is a trend for thresholds for the bluer direction of change to be higher for both yellower and greener directions [compare Figs. 3(a) and 3(b)]. The latter trend (bluer higher than greener) is significant for some combinations of cone complements (see Appendix A). This result suggests that a weak bias for daylight illuminations may be preserved in the appearance level color constancy mechanisms of CVDs. If the bias is weaker in this population, we may expect to see a bias for bluer illuminations and not yellower as the bias also seems to be stronger for daylight changes in the bluer direction in normal trichromats [10–13].

### D. Effects of Dimensionality of Color Vision

**Fig. 4.**
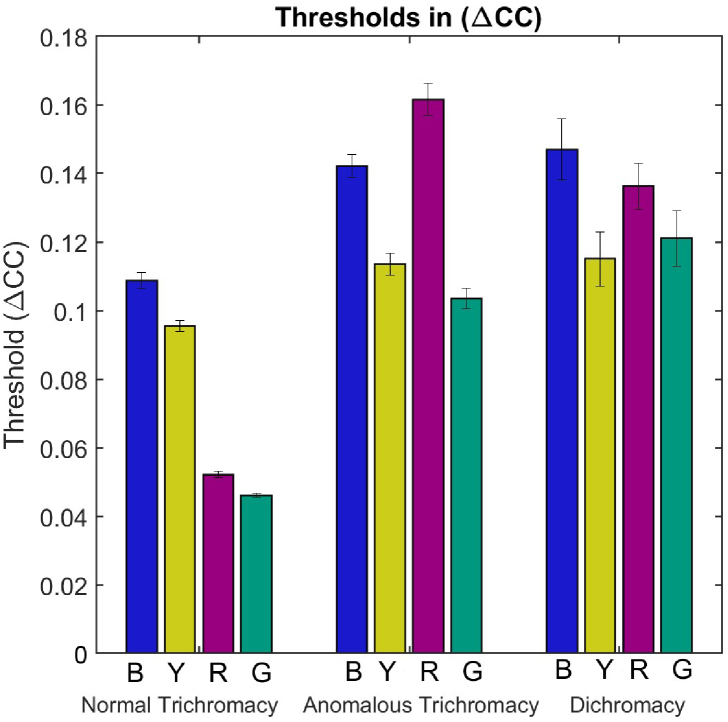
Illumination discrimination thresholds across the four directions of illumination change (B, blue; Y, yellow; R, red; G, green) split by color-vision type (normal trichromat versus anomalous trichromat versus dichromat) expressed as total cone contrast ( ΔCC ). Error bars are ±1SEM .

A further question is whether illumination discrimination ability for daylight changes is better preserved in CVDs who maintain three-dimensional color vision (anomalous trichromats) than CVDs whose color vision is reduced to two dimensions (dichromats). Therefore, we also analyze the ΔCC thresholds with observers split into three groups. A one-way repeated measures ANOVA on the ΔCC thresholds with a between groups factor of color-vision type (three levels: normal versus anomalous trichromat versus dichromat) and a within groups factor of direction of illumination change (four levels: bluer, yellower, redder, greener) finds a significant interaction effect of color-vision type and direction of illumination change on thresholds [ F(6,123)=6.6 , p<.001 ; Fig. 4].

Simple main effects of color-vision type reveal that the interaction effect is driven by significantly larger thresholds for the anomalous trichromats and dichromats in the redder [ F(2,41)=27.8 , p<.001 ] and greener [ F(2,41)=17.6 , p<.001 ] directions of illumination change than normal trichromats, but no difference across groups for the bluer [ F(2,41)=2.39 , p=.0.42 ] and yellower [ F(2,41)=0.98 , p=1.53 ] directions.

The simple main effect of the direction of illumination change is significant for normal trichromats and anomalous trichromats, but not dichromats [normal trichromats, F(3,63)=31.9 , p<.001 ; anomalous trichromats, F(3,39)=5.07 , p=.05 ; dichromats, F(3,21)=1.26 , p=.94 ]. In the normal-trichromat group, thresholds are significantly higher in the bluer and yellower directions of change than in the redder and greener directions of change ( p<.001 in all cases). For anomalous trichromats, thresholds tend to be higher in the bluer direction than in the greener direction ( p=.09 ). In short, these results suggest that the illumination discrimination ability for daylight changes is preserved equally well in both anomalous trichromats and dichromats. They also suggest that the weakened daylight bias we hypothesized above may only be present in anomalous trichromats and not dichromats, as there is no simple main effect of illumination in the latter group. However, this likely reflects the smaller sample size and, hence, reduced power in this group for detecting a weakened bias.

## 4. DISCUSSION

The question is still open as to the appropriate measure for quantifying illumination discrimination performance across individuals with different color-vision types, ranging from normal and anomalous trichromacy to dichromacy. To compare performance between groups, it is essential to use the same objective metric to parametrize the stimuli. Within individuals, to compare performance across different chromatic directions, it is important to use a metric that is uniform across the individual’s color space. Here, we use standard CIELUV space to compare performance between normal trichromacy versus X-linked CVD, but choose a cone-contrast metric to compare performance between chromatic directions, tailored to the cone complements of different color-vision types.

The pattern of relative thresholds across different chromatic directions for normal trichromats is the same using the two different metrics, with thresholds for bluer and yellower changes higher than thresholds for redder or greener changes, suggesting a “daylight bias” for appearance level color constancy. For individuals with CVD, the pattern of relative thresholds differs between the two measures. In the CIELUV metric, thresholds are significantly lower in the bluer and yellower directions than the redder direction and significantly lower in the yellower direction than the greener direction, suggesting the opposite of a daylight bias (which we previously reported [31]). In the cone-contrast metric, the pattern instead suggests a weak daylight bias, with thresholds in the bluer direction higher than in the greener direction. Importantly, the pattern of relative thresholds across chromatic directions is essentially the same for all combinations of sets of cone-contrast measures calculated for each color-vision type, using different pairs of L and M cone varieties specified by their peak sensitivity wavelengths. The trend towards higher bluer thresholds is significant in some cone combinations for anomalous trichromats. With the cone-contrast measure, there is also a trend for redder thresholds to be higher than greener thresholds, which is also significant for some cone complement combinations in anomalous trichromats.

Cone contrasts provide an estimate of the lowest level signal on which visual discrimination is based, but they likely do not fully determine appearance. A computational-observer analysis based on a physiologically realistic model of early human vision indicates that although cone excitations alone in normal trichromats are sufficient to predict elevated blue thresholds on IDTs, relative to red and green, they also predict elevated thresholds for the yellow direction [32], which do not occur in previous studies [10–13]. The analysis suggests that post-cone-isomerization processes further influence performance on IDTs.

The elevation of thresholds in the red and green directions that we find for both dichromats and anomalous trichromats, relative to normal trichromats, is also consistent with the predictions of the computational-observer model tailored for protanopes (but not deuteranopes). We did not analyze data for protanopes and deuteranopes separately here due to small sample sizes, so we cannot say whether the elevation of thresholds that we measured in the red and green directions for dichromats and anomalous trichromats is driven by the protanopes. However, we emphasize that it is not only cone varieties, as considered in the cone-contrast metric analysis, but also L:M cone ratios that vary greatly across individuals [33]. Ding *et al*. [32] considered only the CIE 2006 standard cone absorption spectra, and all L:M cone ratios were roughly 2:1.

Including both typical (bluer–yellower) and atypical (redder–greener) illumination changes as stimuli, and using the cone-contrast metric to compare performance across these directions, enables us to substantially advance earlier findings ([16]) for illumination discrimination in CVD. Lower thresholds on the IDT indicate higher sensitivity to changes in scene appearance caused by changes in illumination and, therefore, we suggest, poorer color constancy. Given that CVDs have higher thresholds than normal trichromats for illumination changes in the red and green directions, may we conclude that they have better color constancy for these directions? In one sense, yes, and in another, no. Scene appearance would appear more stable under these illumination changes. Yet, we would predict that discrimination of objects across different scenes lit by illuminations that differ in red or green directions would be poorer than for normal trichromats. Crucially, in both metrics, discrimination of illumination changes in the blue and yellow directions, which more closely resemble natural daylight changes, is not significantly worse for CVDs compared with normal trichromats. Color constancy for natural daylight changes is preserved.

**Table 1.** Maximum Luminance Difference (Diff) across Each Direction of Illumination Change, Calculated as Difference in Luminance between Reference Illumination (Ref lum) and Furthest Comparison Illumination (Far lum), for Two Combinations of [M, L] Cone Varieties (Specified by Peak Sensitivity Wavelengths) for Each of Five Color-Vision Types [Normal-Trichromatic (NT), Protanopic (PD), Deuteranopic (DD), Protanomalous (PA), and Deuteranomalous (DA)]

CVD Type	NT1	NT2	PD1	PD2	DD1	DD2	PT1	PA2	DD1	DA2
[M, L]	530	536	530	536	549	559	530	533	549	556.5
Cone Pairs	559	549	530	536	549	559	536	536	559	559
***“bluer” direction***										
**Ref lum**	52.84	52.56	53.46	52.89	51.89	51.61	53.27	53.08	51.71	51.62
**Far lum**	57.06	56.61	59.01	57.55	54.76	53.22	58.52	58.03	53.73	53.33
**Diff**	−4.22	−4.05	−5.54	−4.66	−2.86	−1.61	−5.24	−4.95	−2.03	−1.71

***“yellower” direction***										
**Ref lum**	53.01	52.71	53.62	53.04	52.06	51.80	53.43	53.23	51.89	51.81
**Far lum**	48.34	48.14	47.49	47.81	48.80	50.03	47.60	47.70	49.61	49.91
**Diff**	4.67	4.57	6.13	5.23	3.26	1.77	5.83	5.53	2.27	1.90

***“redder” direction***										
**Ref lum**	52.78	52.50	53.41	52.84	51.83	51.54	53.22	53.03	51.64	51.55
**Far lum**	50.32	49.72	49.84	49.58	49.98	51.26	49.76	49.65	50.83	51.12
**Diff**	2.46	2.79	3.57	3.25	1.86	0.28	3.46	3.38	0.81	0.42

***“greener” direction***										
**Ref lum**	52.76	52.49	53.40	52.82	51.81	51.51	53.20	53.01	51.61	51.52
**Far lum**	54.82	54.83	56.45	55.60	53.32	51.58	56.17	55.90	52.16	51.72
**Diff**	−2.06	−2.35	−3.06	−2.77	−1.50	−0.07	−2.96	−2.89	−0.55	−0.20

**Table 2.** Results from Statistical Analyses of Variations in Thresholds for Illumination Change Discrimination across Chromatic Directions, Calculated Using DeMarco Cone Fundamentals for Five Color-Vision Types [Normal-Trichromatic (NT), Protanopic (PD), Deuteranopic (DD), Protanomalous (PA), and Deuteranomalous (DA)]^*a*^

CVD Type	NT	PD	DD	PA	DA

[M, L]	543	543	566	543	558
Cone Pairs	566	543	566	553	566
**Two Group Analysis: NT versus X-Linked CVD**					
Main interaction	CV type x direction		F(3,126)=9.01		p<0.001
Simple main effects	CV type (2 groups)				
	B: NT = CVD		t(42)=−2.25		p=0.119
	Y: NT = CVD		t(42)=−1.46		p=0.606
	R:NT<CVD		t(42)=−7.22		p<.001
	G:NT<CVD		t(42)=−5.80		p<.001
Simple main effects	Direction				
	**NT**		F(3,63)=33.63		p<.001
	NT:B>R				p<.001
	NT:B>G				p<.001
	NT:Y>R				p<.001
	NT:Y>G				p<.001
	**CVD**		F(3,63)=6.37		p=.007
	CVD:B>G				p<.007
	CVD:R>G				p<.045

**Three Group Analysis: NT versus AT versus Di**					
Main interaction	CV type x direction		F(6,123)=5.11		p<0.001
Simple main effects	CV type (3 groups)				
	B: NT = AT = Di		F(2,41)=2.48		p=.386
	Y: NT = AT = Di		F(2,41)=1.04		p=1.446
	R:NT<AT,Di		F(2,41)=27.35		p<.001
	G:NT<AT,Di		F(2,41)=17.39		p<.001
Simple main effects	Direction				
	**NT**		F(3,63)=33.63		p<.001
	NT:B>R				p<.001
	NT:B>G				p<.001
	NT:Y>R				p<.001
	NT:Y>G				p<.001
	**AT**		F(3,39)=5.19		p=.044
	AT:B>G				p=0.017
	**Di**		F(3,21)=1.76		p=.656

^*a*^ [M, L] cone fundamentals are specified by peak sensitivity wavelengths. AT, anomalous trichromacy; Di, dichromacy.

**Table 3.** Results from Statistical Analyses of Variations in Thresholds for Illumination Change Discrimination across Chromatic Directions, Calculated Using a Different Combination of Cone Pairs (cf. Table 2 and Main Text) for Five Color-Vision Types [(Normal-Trichromatic (NT), Protanopic (PD), Deuteranopic (DD), Protanomalous, (PA), and Deuteranomalous (DA)]^*a*^

CVD Type	NT2	PD2	DD2	PA2	DA2

[M, L]	536	536	559	533	556.5
Cone Pairs	549	536	559	536	559
**Two Group Analysis: NT versus X-Linked CVD**					
Main interaction	CV type x direction		F(3,126)=11.743		p<0.001
Simple main effects	CV type (2 groups)				
	B: NT = CVD		t(42)=−2.22		p=0.128
	Y: NT = CVD		t(42)=−1.43		p=0.64
	R:NT<CVD		t(42)=−7.28		p<.001
	G:NT<CVD		t(42)=−5.83		p<.001
Simple main effects	Direction				
	**NT**		F(3,63)=31.63		p<.001
	NT:B>R				p<.001
	NT:B>G				p<.001
	NT:Y>R				p<.001
	NT:Y>G				p<.001
	**CVD**		F(3,63)=5.77		p=.011
	CVD: B = G				p=0.071
	CVD:R>G				p<.048

**Three Group Analysis: NT versus AT versus D**					
Main interaction	CV type x direction		F(6,123)=6.63		p<0.001
Simple main effects	CV type (3 groups)				
	B: NT = AT = Di		F(2,41)=2.41		p=.41
	Y: NT = AT = Di		F(2,41)=0.999		p=1.51
	R:NT<AT,Di		F(2,41)=27.81		p<.001
	G:NT<AT,Di		F(2,41)=17.58		p<.001
Simple main effects	Direction				
	**NT**		F(3,63)=33.63		p<.001
	NT:B>R				p<.001
	NT:B>G				p<.001
	NT:Y>R				p<.001
	NT:Y>G				p<.001
	**AT**		F(3,39)=5.08		p=.05
	AT: B = G				p=0.09
	**Di**		F(3,21)=1.28		p=.922

^*a*^ [M, L] cone fundamentals are specified by peak sensitivity wavelengths. AT, anomalous trichromacy; Di, dichromacy.

## Data Availability

Data underlying the results presented in this paper will be made available upon request to the authors.

## APPENDIX A

Calculations of luminance and cone contrasts depend on the assumed spectral sensitivities of the M and L cone types, which are known to vary, both in normal trichromacy and anomalous color vision. As described in the main text, the relevant calculations and subsequent statistical tests have been carried out for different combinations of [M,L] peak spectral sensitivities and examples are reported in Tables  1–3.
